# Pericardial tamponade due to haemorrhagic pericardial effusion as a complication of prasugrel: a case report

**DOI:** 10.1186/s12872-016-0338-8

**Published:** 2016-08-30

**Authors:** Fathima Aaysha Cader, M. Maksumul Haq, Sahela Nasrin, Md. Rezaul Karim

**Affiliations:** 1 Department of Cardiology, Ibrahim Cardiac Hospital & Research Institute (ICHRI), Shahbagh, Dhaka, 1000 Bangladesh; 2 National Institute of Cardiovascular Diseases, Dhaka, Bangladesh

**Keywords:** Cardiac tamponade, Haemorrhage, Prasugrel, Case report

## Abstract

**Background:**

Striking an adequate balance between bleeding risks and prevention of stent thrombosis can be challenging in the setting of percutaneous coronary intervention (PCI) with drug eluting stents (DES) in acute myocardial infarction (MI). This is more pronounced in patients treated with both low molecular weight heparin (LMWH) and dual antiplatelet therapy (DAPT). Prasugrel, a second generation thienopyridine with more potent platelet inhibition capability, is associated with significant bleeding risks. This risk of bleeding is often underestimated when prescribing pharmacological agents such as DAPT and LMWH, designed to reduce ischaemic events following PCI in acute MI. Life-threatening haemorrhagic pericardial and pleural effusions not associated with access site bleeding are a rare example of such bleeding complications.

**Case presentation:**

We report a case of a Bangladeshi male who developed cardiac tamponade resulting from haemorrhagic pericardial effusion as well as bilateral pleural effusions, 9 days after PCI with a DES, while on prasugrel and aspirin. He had presented late with inferior ST elevation myocardial infarction (STEMI), and was therefore also given enoxaparin initially. Haemorrhagic pericardial and pleural fluid were drained, and the patient was discharged on DAPT comprising of aspirin and clopidogrel. Following PCI to obtuse marginal, which was done as a staged procedure 6 months later, he was commenced on ticagrelor instead of clopidogrel. He developed no further bleeding complications over 1 year of follow up.

**Conclusion:**

Non-access site bleeding such as this, leading to haemorrhagic pericardial and pleural effusions can be rare and life-threatening. Furthermore, patients with acute coronary syndromes (ACS) have marked variation in their risk of major bleeding. Since haemorrhagic complications are associated with mortality, maintaining a balance between the risk of recurrent ischemia and that of bleeding is of paramount importance. The use of validated bleeding risk scores, careful monitoring of patients on DAPT with LMWH, or a switch over to agents with lesser risk of bleeding may reduce such complications.

## Background

Patients admitted with acute myocardial infarction (MI) are at considerable risk of ischemic complications particularly during the acute phase, warranting aggressive pharmacological and interventional therapies. A combination of Dual Antiplatelet therapy (DAPT) and anticoagulation is commonly prescribed, particularly in the setting of percutaneous coronary intervention (PCI). However, the same interventions designed to protect against such ischemic complications are responsible for the increased likelihood of major bleeding during hospitalization [1]. Currently, newer generation thienopyridine antiplatelet agents, such as prasugrel are frequently used in combination with aspirin, particularly in the setting of percutaneous coronary intervention (PCI) in acute coronary syndromes (ACS) [1]. This is especially significant with drug eluting stent (DES) implantation, where adequate inhibition of platelet aggregation is paramount to preventing stent thrombosis. Prasugrel, an irreversible platelet P2Y12 receptor inhibitor has superior platelet inhibition properties, but is associated with a greater risk of haemorrhagic manifestations in comparison to clopidogrel or ticagrelor [2, 3]. We report the case of cardiac tamponade complicating haemorrhagic pericardial effusion in addition to haemorrhagic pleural effusions in a patient who was concomitantly administered aspirin, prasugrel and low molecular weight heparin (LMWH).

## Case presentation

A 50-year-old Bangladeshi male presented with retrosternal compressive chest pain for 2 days, associated with abdominal discomfort and nausea. He was dyslipidemic, normotensive, diabetic on Insulin, and a recent smoker. On admission his pulse was 110 beats/min, blood pressure was 110/70 mmHg and unremarkable precordial examination. Respiratory rate was 24 breaths/min and chest auscultation revealed bi-basal crackles. Electrocardiography (ECG) showed ST elevation in leads II, III and aVF. Bedside echocardiogram revealed a left ventricular (LV) ejection fraction (EF) of 50 % with hypokinetic basal and mid segments of inferior, infero-septal and infero-lateral walls. Troponin I was raised at 17.46 ng/ml. NT-Pro BNP was 1673.90 pg/ml. Given the delayed presentation (~48 h), he was not thrombolysed, but given loading doses of aspirin (300 mg) and clopidogrel (600 mg), subcutaneous enoxaparin, anti-anginal medications and intravenous furosemide.

**Fig. 1:**
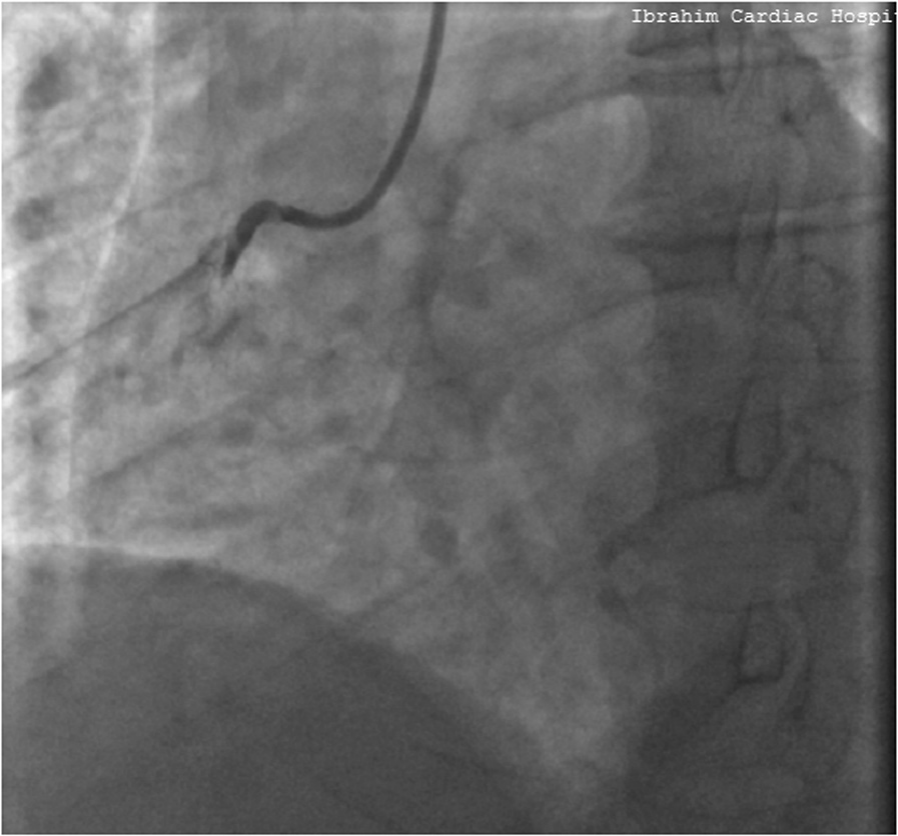
Coronary angiogram showing totally occluded right coronary artery

**Fig. 2:**
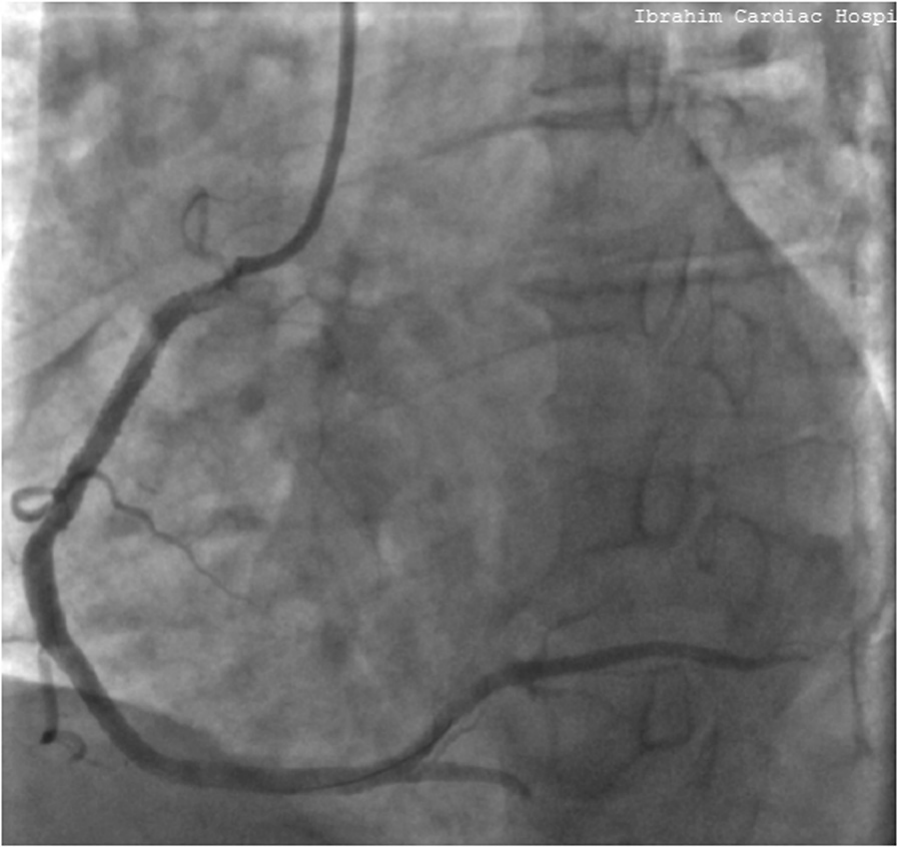
Coronary angiography showing restoration of TIMI III flow following stent implantation in right coronary artery

Coronary angiogram done the same day revealed totally occluded right coronary artery (RCA) (Fig. 1), significantly diseased obtuse marginal (OM) and moderately diseased left anterior descending (LAD) arteries. PCI to RCA (infarct related artery) was done with a 3.5x38mm DES (XIENCE Xpedition Everolimus Eluting Coronary Stent System, Abbott Vascular, USA). Distal Thrombolysis in Myocardial Infarction (TIMI) III flow was achieved with no immediate complication (Fig. 2). As he had received a single dose of LWMH 9 h prior to PCI, he was only given an additional dose of enoxaparin equivalent to 0.3 mg/kg intravenously at the time of PCI, in accordance with guideline recommendations [4]. Unfractionated heparin was not used. Staged PCI to OM was planned for a later date. As he had no contraindications, he was given a loading dose of 60 mg prasugrel, which was continued on 10 mg daily, along with aspirin 75 mg twice daily.

**Fig. 3:**
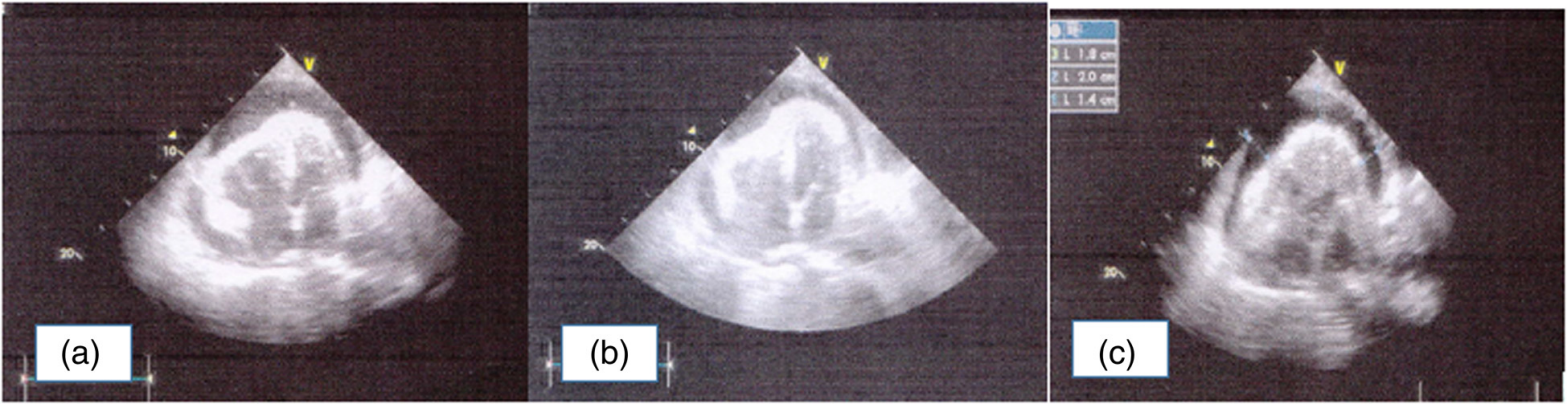
Echocardiography showing massive pericardial effusion with features of diastolic collapse of right ventricle. (**a**) RV in systole (**b**) RV in diastole (**c**) effusion measured at 18 mm, 20 mm, 14 mm surrounding the heart

**Fig. 4:**
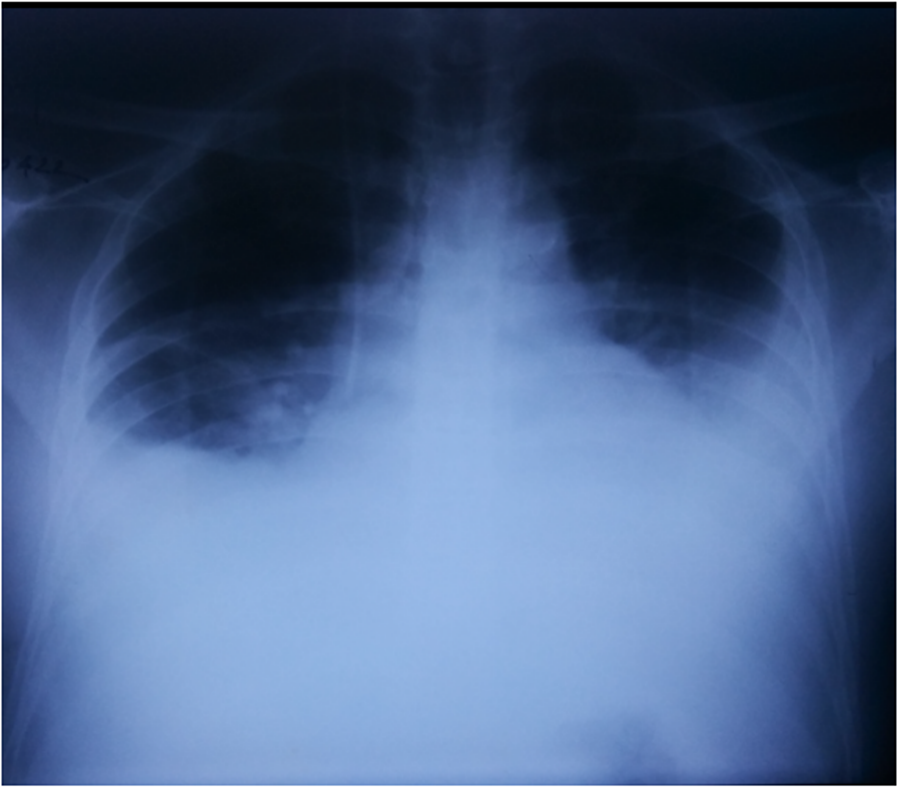
Chest X ray anterior-posterior view (bedside) showing cardiomegaly with evidence of pulmonary oedema and bilateral pleural effusions

Two days after PCI, a pericardial rub was noted. Lung auscultation revealed bi-basal crackles and diminished breath sounds in the lower zones. Mild pericardial effusion (~5 mm) was seen on echocardiogram. As we suspected a haemorrhagic effusion, LMWH was discontinued, prasugrel was withheld, and he was switched back to clopidogrel (75 mg) and reduced dose of aspirin (75 mg) once daily. He was regularly monitored over the next few days, both clinically and echocardiographically: the intensity of the rub gradually diminished, concomitant with increasing pericardial effusion. Serial ECG showed evidence of gradual reduction of voltage. Nine days after PCI, he became hypotensive and tachycardic with jugular venous distension, clinically suggestive of cardiac tamponade; this was confirmed by echocardiography which revealed massive pericardial effusion more in anterior, apical and lateral regions and features of diastolic collapse of right ventricle (Fig. 3), with further reduction of LV EF to 35 %. There was no evidence of left ventricular free wall rupture (LVFWR). Subsequently, he also developed acute pulmonary oedema with bilateral pleural effusions (Fig. 4).

Percutaneous pericardiocentesis was attempted immediately, but as it failed to yield fluid, emergency pericardiotomy was done under general anaesthesia: about 400 ml of haemorrhagic fluid was drained by sub-xiphoid incision. A further 300 ml of blood and 250 ml of clots were evacuated 2 h later through previous subxiphoid incision, by finger dissection from the pericardial cavity, mainly around the RA and RV; a retropericardial drain tube was left in situ, and removed 2 days later. Laboratory examination of the pericardial fluid demonstrated haematocrit value of 40 %, similar to that of peripheral venous blood. Given that the pericardiocentesis was a life-saving procedure that needed to be done on an emergency basis, we proceeded to do so while the patient was still on DAPT, which alternatively, could not be discontinued owing to the impending risk of stent thrombosis.

Post procedure, the central venous pressure reduced to 10cmH_2_0, with improved haemodynamic parameters, and he was extubated the same evening. Following drainage, echocardiography revealed improved EF of 50 % and effusion was reduced to minimal. As his haemoglobin had fallen from 12.9 g/dL to 8.2 g/dl, he was transfused two units of whole blood.

As he remained in acute pulmonary oedema with bilateral pleural effusions, 700 ml of haemorrhagic pleural fluid was aspirated from the left pleural cavity 2 days after pericardial fluid drainage and sent for investigations: pleural fluid protein was 26 mg/dL, LDH raised at 542U/L. ADA was within normal limit, and cytology showed plenty of red blood cells, lymphocytes, histiocytes and mesothelial cells in a proteinaceous background, but no malignant cells. Broad-spectrum intravenous antibiotics were given and he remained afebrile throughout.

Following the resolution of both pericardial and pleural effusions, he was discharged 1 week after pericardiotomy, with stable haemodynamic parameters on DAPT comprising of Aspirin (75 mg) and clopidogrel (75 mg) daily, in addition to diuretics and anti-ischaemic medication. He underwent successful staged PCI to OM 6 months later, at which time a check angiography of the left system revealed mild in-stent restenosis of the RCA stent, which was treated with a balloon angioplasty with satisfactory result. Subsequently, he was given DAPT comprising of Aspirin 75 mg daily and ticagrelor 90 mg twice daily. He remained well at 1 year of follow up with no further effusions, as confirmed by echocardiography.

## Discussion

Pericardial effusions are found in 15 to 28 % of patients presenting with a 1st myocardial infarction. [5–8] They are more common in patients with anterior STEMI, larger infarcts and in the presence of congestive heart failure [5, 8, 9]. It usually appears during the initial 5 days, disappearing slowly over during several weeks to months [6]. Two-thirds of those patients with moderate-to-severe effusions develop tamponade or left ventricular free wall rupture (LVFWR) [10, 11].

Haemorrhagic pericardial effusion and cardiac tamponade in the setting of Acute MI and PCI in this patient may have arisen due to three principal causes: coronary artery perforation during PCI, haemorrhagic pericarditis, and cardiac rupture, most often LVFWR.

The frequency of iatrogenic coronary perforations has increased over the years, with the advent of new percutaneous revascularization techniques and greater incidence of PCI [9, 10]. They are usually associated with guide wire trauma or catheter manipulation of atherosclerotic vessel segments during PCI [9, 10].

In our case, a review of the angiographic images from various planes both during and after completion of the procedure, as well as a retrospectively after the effusion developed, showed no angiographic evidence of perforation, i.e. an extraluminal crater, contrast extravasation or cavity spilling after stenting. Moreover, it is Ellis Type III perforation (defined as extravasation through frank (>1 mm) perforation) that is associated with the rapid development of cardiac tamponade (42.9 %) [9], which would usually have been immediately evident during procedure. Furthermore, frank perforation leading to haemorrhagic pericardial effusion and cardiovascular collapse is usually known to occur as early as 24 h after PCI [9, 10, 12, 13], whereas cardiac tamponade in our patient developed approximately 8 days after PCI and only after the commencement of prasugrel. Nevertheless, a small perforation, not visible initially, cannot be completely excluded; it may have begun to leak seriously following the onset of action of prasugrel, eventually resulting in the large haemorrhagic pericardial effusion.

Previously, there have been cases described in the literature of tamponade arising from haemorrhagic pericarditis associated with thrombolysis, usually associated with anterior MI [14] and more recently with the use of glycoprotein IIb/IIIa inhibitors during PCI [13, 15] and combinations thereof [16]. However, few cases of tamponade attributed to prasugrel-induced haemorrhagic effusions have been reported in the literature.

Prasugrel, an irreversible P2Y12 receptor blocker, when given in the background of PCI, has been associated with significantly reduced rates of ischaemic events i.e. nonfatal MI & stent thrombosis; however, these benefits were limited by higher rates of TIMI major bleeding (2.2 %), both life-threatening (1.3 %) & fatal bleeding (0.3 %) as compared to clopidogrel (0.8 and 0.1 % respectively) [2].

Alternatively, clopidogrel resistance is an important issue that needs to be considered when prescribing antiplatelet agents post-PCI in Bangladeshi subjects. A pioneer study conducted in our centre using the VerifyNow ® system found that nearly half (46.7 %) of the patients in the clopidogrel group were resistant to the drug as opposed to none in the prasugrel group [17]. Moreover, guidelines recommend that a loading dose of 325 mg of non-enteric aspirin be given prior to PCI [4]. Non-enteric aspirin is not commercially available in Bangladesh; enteric aspirin has been attributed as a cause of aspirin resistance, due to reduced bio-availability from impaired absorption and inadequate platelet inhibition due to uninhibited platelet cyclooxygenase activity [18, 19]. For this reason, when prescribed with clopidogrel, our centre follows a practice of an initial maintenance dose of aspirin 75 mg twice daily, followed by a reduction to 75 mg daily later on.

The CRUSADE Bleeding Score calculated for this patient was 26, corresponding to an estimated low risk of bleeding, and a 6.3 % estimated risk of in-hospital major bleeding [20]. This patient’s calculated ACUITY-HORIZONS-AMI Integer-Based Risk Score was 11, corresponding to a moderate category with a 3.3 % risk of Non–coronary artery bypass graft (CABG)-Related Major Bleeding Within 30 days of Patient Presentation with ACS [21]. Clinical trial data show that complications with prasugrel were more frequent in specified high risk subgroups (age >75 years, previous stroke/ transient ischaemic attack (TIA), body weight <60 kg), none of which our patient had [2].

Thus, given this relative lower pre-procedural bleeding risk, and absence of contraindications such as prior stroke or TIA, we opted for prasugrel as per guidelines, once the coronary anatomy was known and PCI to infarct-related artery was planned [1]. Our patient was initially given a clopidogrel load as part of guideline-recommended antiplatelet therapy in ACS prior to PCI; a further rationale for this strategy was that, should the angiography reveal coronary artery disease requiring CABG, clopidogrel need only be stopped 5 days or lesser before CABG, as opposed to prasugrel, whose duration of action is longer, requiring it to be withheld 7 days before CABG. Moreover, off pump CABG may be considered within 24 h of clopidogrel or ticagrelor administration [1].

In clinical practice, most bleeding complications observed have been minor and related to the angiographic access site leading to haematoma or mucosal bleeding in the form of epistaxis or gum bleeding. Although prasugrel has been known to increase bleeding complications [2], the association with haemorrhagic pericardial effusion leading to tamponade has been rarely reported. However, the administration of a loading dose of prasugrel following the previous loading dose of clopidogrel could be an additional factor for excess bleeding, although such a complication has not been observed previously in our centre.

Kaul et al. who conducted a multicentric, hospital registry in India of 1000 patients with ACS undergoing PCI who were administered prasugrel found only one patient who developed hemorrhagic pericardial effusion following 2 days post PCI which required surgical drainage for resulting tamponade [21]. This patient had undergone delayed angioplasty following a STEMI, just like our case. This also poses a possible association of haemorrhagic infarction and subsequent haemorrhagic pericarditis due to late reperfusion therapy which may be considered [13], as symptom onset to balloon time was nearly 60 h in our case, owing to delayed presentation. In fact, among the predictors of cardiac tamponade in STEMI patients are lack of reperfusion therapy and late hospital admission [22], both of which corresponds with our patient, the other factors being lateral site, increasing age and increasing number of leads involved [22]. This compounded by the potent anti-platelet properties of prasugrel and as such, the excessive bleeding tendency was the most likely mechanism for this devastating complication. Dressler’s syndrome which usually presents with fever, pleuro-pericardial discomfort, friction rub, leucocytosis, raised ESR and pulmonary infiltrates typically presents weeks to months after acute MI, is recurrent and causes a fibrinous pericarditis [2, 23]. Although the timing of effusions in our patients is slightly earlier than those described for Dressler’s syndrome, the possibility of an inflammatory process should be considered, which may have eventually led to haemorrhagic effusions, owing to the effect of the strong antiplatelet action.

Our patient was also administered LMWH, which was discontinued as soon as the mild effusion was noted. Combination of LWMH with prasugrel could be associated with increased bleeding risks. As such, detection of pericardial effusion >1 cm on echocardiography or enlarging pericardial effusion is an indication for discontinuation of anticoagulation unless continuation is strongly indicated, in which case heightened monitoring and observation for signs of possible tamponade is mandatory [2].

Cardiac rupture, particularly LVFWR following acute MI may lead to haemopericardium and cardiac tamponade [24, 25], albeit associated with reduced incidence following revasularisation. Among the two distinct types (i.e. acute (blowout type) and subacute (oozing) type) [25], the subacute free wall rupture may evolve over hours or even days, and presents initially with pericardial effusion-related signs and symptoms [26]. However, this usually occurs 1 to 3 days following acute MI [16], the timing of which is inconsistent with the time course of our patient’s illness. Furthermore, LVFWR can be easily diagnosed echocardiographically by the demonstration of myocardial tear in the LV free wall, low-velocity colour Doppler flow or extravasation of intravenous echocardiographic contrast from LV cavity into the effusion [27], none of which was evident in our patient.

Thus the hypothesis that could explain cardiac tamponade in our patient, in the presence of inferior STEMI implies the association of increased bleeding risks with combined LWMH and DAPT comprising of prasugrel and aspirin. This aetiological association is further supported by the finding of concomitant heart failure and haemorrhagic pleural effusions requiring drainage. Heart failure is known to complicate 49 % of infarctions with pericardial involvement as opposed to 16 % of infarctions without effusion (*p* < 0.01) [3].

This patient developed these life-threatening bleeding complications despite having no contraindication to prasugrel in terms of age, body weight or previous bleeding episodes/stroke. It was also not associated with any ‘warning bleed’ in the form of mucosal of access site haematoma.

Achieving the correct balance between adequate inhibition of platelet aggregation and preventing stent thrombosis on one hand, and the prevention of adverse bleeding outcomes on the other hand, remains a challenging dilemma in the PCI era. The use of validated risk scores for bleeding such as the CRUSADE risk score and the ACUITY-HORIZONS bleeding risk score may be helpful for stratifying and thus identifying patients at increased risk of bleeding complications, and guide the appropriate choice of pharmacotherapy [20, 28]. Furthermore, careful monitoring for potential bleeding risks is imperative when LMWH and DAPT comprising prasugrel is co-administered. This issue is underscored by the fact that complete cessation of antiplatelet drugs could not be afforded at any cost as the patient had already undergone PCI and risked stent thrombosis in that event. Our only option therefore was to switch over to clopidogrel at a lower dose in addition to aspirin, despite the additional issue of clopidogrel resistance in Bangladeshi subjects. Following staged PCI to OM which was done 6 months later, he was started on ticagrelor, which, according to the PLATO trial, has demonstrated improved CV outcomes in comparison to clopidogrel, with no significant difference in the rates of major bleeding similar safety profile with regards to major bleeding, albeit a higher rate of non-CABG related major bleeding (4.5 % for ticagrelor vs. 3.8 % for clopidogrel, *P* = 0.03) [3].

Pericardial effusion can be easily detected by echocardiography and as such close monitoring and routine echocardiographic evaluation of these patients can lead to early detection of a minimal PE and subsequent alteration of drug dosage or switch over to antiplatelet agents of lesser bleeding risk is possible. This case report is a demonstration of how the risk of bleeding in patients is often underestimated, and the need for clinicians to be wary of the ominous signs of life-threatening bleeds occurring due to pharmacotherapy. In addition to strategies for reducing haemorrhagic complications such as the use of newer antithrombotic medications with reduced potential for bleeding and avoidance of overdosing, it is important to identifying patients at risk for major bleeding events by means of risk scores, and prescribe medication accordingly.

## Conclusion

Haemorrhagic complications are an independent risk factor for mortality in patients with ACS and in those undergoing PCI. Patients with ACS have marked variation in their risk of major bleeding. Emphasis is placed on the fact that despite the efficacy of combination therapies comprising antiplatelet agents and anticoagulants in the prevention and treatment of adverse events of coronary interventions in the setting of acute MI, careful monitoring for haemorrhagic complications is paramount. Routine evaluation of patients at risk for excessive bleeding by means of bleeding risk scores are recommended. A cessation of the offending agent or switch over to drugs with lesser bleeding risks may be warranted.

## Abbreviations

ACS: Acute coronary syndrome
DAPT: Dual antiplatelet therapy
DES: Drug eluting stents
ECG: Electrocardiogram
EF: Ejection fraction
LAD: Left anterior descending
LMWH: Low molecular weight heparin
LV: Left ventricular
LVFWR: Left ventricular free wall rupture
MI: Myocardial infarction
OM: Obtuse marginal
PCI: Percutaneous coronary intervention
RCA: Right coronary artery
STEMI: ST elevation myocardial infarction
TIMI: Thrombolysis in myocardial infarction
